# The Cardioprotective Effects of Adiponectin in Diabetes

**DOI:** 10.1007/s11892-025-01610-5

**Published:** 2025-10-13

**Authors:** Tara Kazemi, Yuanjie Mao, Tina Zhang

**Affiliations:** 1 Ohio University Heritage College of Osteopathic Medicine, Athens, OH 45701 USA; 2 Diabetes Institute, Ohio University, Athens, OH 45701 USA; 3 Endocrinology & Diabetes Clinic, OhioHealth Castrop Health Center, Athens, OH 45701 USA; 4 Department of Biological Sciences, University of Pittsburgh, Pittsburgh, PA 15213 USA

**Keywords:** Adiponectin, Diabetes mellitus, Complications, Congestive heart failure

## Abstract

**Purpose of Review:**

Adiponectin, a hormone secreted by adipocytes, plays a crucial role in maintaining metabolic balance and supporting cardiovascular health. Although it is known for its protective effects, such as improving insulin sensitivity, reducing inflammation, and maintaining endothelial function, there are paradoxical associations between high adiponectin levels and increased cardiovascular mortality—referred to as the “adiponectin paradox”—which complicates its clinical interpretation. This review explores the cardioprotective effects of adiponectin in both type 1 and type 2 diabetes, focusing on its potential to regulate glucose metabolism and prevent cardiovascular complications.

**Recent Findings:**

By reviewing key studies, the article evaluates adiponectin’s diverse roles and compares its effects on cardiovascular outcomes across diabetes subtypes, especially in diabetic cardiomyopathy, with an emphasis on congestive heart failure.

**Summary:**

The findings underscore the importance of further research into therapeutic strategies aimed at modulating adiponectin levels, particularly for individuals with diabetes and congestive heart failure. Understanding the dual nature of adiponectin’s effects is critical for developing target interventions to improve cardiovascular outcomes in diabetic populations.

## Introduction

Adiponectin, a hormone produced by adipocytes, plays a crucial role in maintaining metabolic balance and promoting cardiovascular health. It improves insulin sensitivity, enhances glucose uptake, and reduces pro-inflammatory pathways, thereby offering protection against complications associated with diabetes [1–3]. Previous studies have found that higher adiponectin levels are associated with better metabolic health, particularly in lean individuals, while lower levels are linked to insulin resistance, systemic inflammation, and a higher risk of cardiovascular complications [1–3]. However, in certain populations—such as those with advanced heart failure—elevated adiponectin levels paradoxically correlate with worse cardiovascular outcomes, a phenomenon known as the “adiponectin paradox” [4, 5].

Cardiovascular complications remain the leading cause of death in individuals with type 1 diabetes (T1D) and type 2 diabetes (T2D). Adiponectin’s role across these subtypes reflects the distinct pathophysiologies of each. The complexity of adiponectin’s dual role in metabolic and cardiovascular health has attracted significant research attention, particularly in the context of diabetes. While it is known that adiponectin levels differ between T1D and T2D, the mechanisms underlying these differences and their impact on cardiovascular health are poorly understood [3]. This review explores adiponectin’s regulatory mechanisms, its paradoxical associations, and the implications for therapeutic strategies, synthesizing key findings to better understand its dual role in diabetes and cardiovascular health.

## Adiponectin in Diabetes

**Fig. 1:**
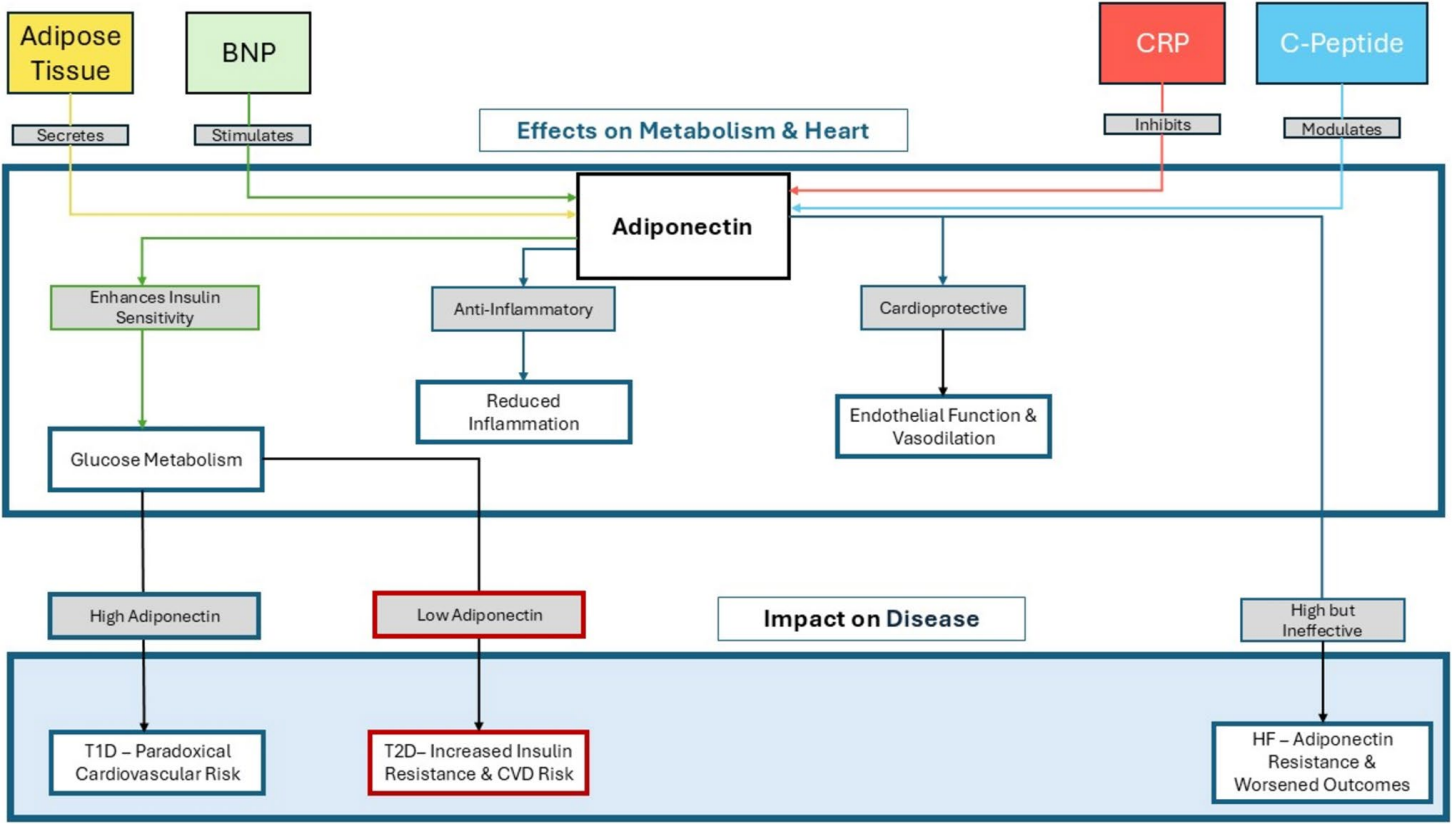
The major regulators of adiponectin, the effects of adiponectin on metabolism and cardiovascular health, and the implications of adiponectin in diabetes and heart failure. BNP, brain natriuretic peptide; CRP, C-reactive protein; CVD, cardiovascular diseases; HF, heart failure; T1D, type 1 diabetes; T2D, type 2 diabetes

Adiponectin plays a central role in metabolic regulation by activating several downstream pathways, notably the peroxisome proliferator-activated receptor α (PPARα) and 5’ AMP-activated protein kinase (AMPK) signaling pathways [6]. These pathways mediate adiponectin’s effects on glucose homeostasis, lipid metabolism, and cardiovascular protection, positioning it as a critical target in the management of diabetes and other metabolic disorders [6]. Furthermore, adiponectin’s regulatory mechanisms are shaped by complex interactions involving C-peptide levels, adipose tissue quantity and quality, insulin resistance, and sex hormones (Fig. 1), highlighting its multifaceted role in metabolic health.

### Regulatory Pathways

#### Adiponectin Receptors

Adiponectin exerts its effects mainly through two receptors: AdipoR1 and AdipoR2, which are broadly expressed in metabolic tissues such as skeletal muscle, liver, and heart [1, 3]. These receptors mediate adiponectin’s activation of critical downstream signaling pathways, especially AMPK and PPARα, which are central to regulating glucose and lipid metabolism [1, 7]. AdipoR1 is primarily found in skeletal muscle, where it activates the AMPK signaling pathway, enhancing glucose uptake, fatty acid oxidation, and mitochondrial biogenesis [8]. Meanwhile, AdipoR2, which is highly expressed in the liver, activates PPARα, promoting fatty acid oxidation, reducing triglyceride accumulation, and improving hepatic insulin sensitivity [7].

The function and expression of these receptors are key to adiponectin’s beneficial metabolic effects. However, their activity is often diminished in metabolic disorders like T2D and obesity. In T2D, receptor expression is significantly reduced, impairing adiponectin signaling. For example, a 40% reduction in AdipoR1 expression in the skeletal muscle of T2D patients leads to decreased AMPK activation and reduced glucose uptake [9]. Similarly, AdipoR2 expression is also notably lower in insulin-resistant individuals, contributing to increased triglyceride accumulation and worsened lipid dysregulation in the liver [3]. Adiponectin resistance may also result from dysfunction in the T-cadherin receptor, which typically anchors high-molecular-weight (HMW) adiponectin to cellular membranes, facilitating protective downstream actions like reducing ceramide accumulation and improving insulin sensitivity [6]. Impaired T-cadherin binding can increase the activity of glycosylphosphatidylinositol-specific phospholipase D (GPI-PLD), which disrupts the receptor’s function, leading to elevated circulating adiponectin levels but reduced biological activity [10]. In advanced metabolic or cardiovascular diseases, chronic inflammation, oxidative stress, and receptor dysfunction further impair adiponectin signaling. Resistance in the AMPK pathway, a crucial mediator of adiponectin’s metabolic benefits, can limit its capacity to improve glucose uptake, enhance fatty acid oxidation, and reduce oxidative stress [6, 11].

#### Adipose Tissue

Adiponectin secretion is closely linked to both the quantity and quality of adipose tissue. Healthy adipose tissue secretes adiponectin to enhance insulin sensitivity, regulate glucose tolerance, promote fat metabolism, and maintain metabolic homeostasis, offering protection against diabetes [12]. HMW adiponectin, which is predominantly secreted by healthy adipose tissue, shows an inverse relationship with visceral fat and markers of adipose dysfunction, such as hypoxia, oxidative stress, and inflammation [6, 13]. Visceral fat accumulation is strongly associated with lower adiponectin levels, with studies reporting correlations as high as *r* = −0.57 (*P* < 0.001), highlighting the detrimental impact of unhealthy adipose tissue on adipokine regulation [14]. Chronic inflammation within adipose tissue, driven by cytokines like tumor necrosis factor (TNF-α) and interleukin-6 (IL-6), downregulates adiponectin gene expression and disrupts adipocyte function, leading to reduced circulating adiponectin levels [15]. Additionally, oxidative stress impairs adipose tissue function, contributing to adipose tissue fibrosis and a further reduction in adiponectin secretion [10]. Therefore, adiponectin secretion serves as an important indicator of both the quantity and quality of adipose tissue, making it a key marker of metabolic health.

#### Insulin Secretion

C-peptide, a marker of pancreatic beta-cell function and insulin secretion, plays a significant role in regulating adiponectin production, with notable differences between T1D and T2D [14, 16]. In T1D, where beta-cell function is severely compromised, C-peptide levels are markedly reduced [16]. This lack of C-peptide leads to increased adiponectin production in individuals with T1D, as C-peptide typically serves as an inhibitory regulator of adipocyte activity [14].

#### Age

In elderly populations, particularly those over the age of 65, adiponectin levels often rise [17, 18]. However, this increase does not always correlate with improved metabolic health [17, 18]. Instead, it has been associated with a phenomenon known as “adiponectin resistance” [4]. In this state, elevated adiponectin levels fail to produce the usual protective effects, possibly due to impaired receptor function or dysfunction in downstream signaling pathways [19]. This paradox may help explain why older individuals with high adiponectin levels remain at increased risk for cardiovascular disease and metabolic complications, despite the hormone’s beneficial effects in younger populations.

#### Sex Hormones

Sex differences in adiponectin levels are well-established, with women generally exhibiting higher levels than men [12, 14, 17, 20, 21]. This difference is largely attributed to the influence of estrogen, which promotes the production of HMW adiponectin, the most biologically active isoform [12, 17]. HMW adiponectin contributes to better metabolic outcomes in women, including improved insulin sensitivity and a more favorable lipid profile, characterized by higher levels of high-density lipoprotein (HDL) cholesterol and lower triglyceride levels [17]. In contrast, androgens such as testosterone appear to suppress adiponectin production. A previous study found that testosterone inhibits adiponectin production in adipocytes, which likely contributes to the lower circulating adiponectin levels observed in men compared to women [21]. This hormonal difference may partially explain why men often experience earlier onset and more severe insulin resistance and cardiovascular complications.

Age also plays a significant role in the relationship between sex hormones and adiponectin levels, particularly in women. A comparison of adiponectin and HDL cholesterol levels by age (with 50 years as the cutoff) revealed a marked decline in both adiponectin and HDL cholesterol in women aged 50 and older, coinciding with reduced estrogen levels during menopause [22]. A study suggests that fluctuations in estrogen levels affect not only adiponectin production but also its structure and isoform balance [23]. Early postmenopausal women show a shift toward lower HMW adiponectin concentrations, which reduces its biological activity [23]. This change may exacerbate age-related metabolic dysfunction, such as increased visceral fat, lower HDL cholesterol, and higher triglycerides, all of which contribute to insulin resistance and cardiovascular risk [24]. In contrast, men of the same age exhibit increase in both adiponectin and HDL cholesterol, likely due to declining testosterone levels [14]. These findings further support the protective role of estrogen in regulating adipokine production and metabolic health, suggesting the decline in adiponectin levels in postmenopausal women as a contributing factor to age-related metabolic disorders [22, 24].

### Adiponectin in Type 1 Diabetes

Numerous studies have shown that serum adiponectin levels are consistently elevated in individuals with T1D compared to healthy controls [7, 12, 25, 26]. For instance, a cohort of 1393 participants revealed significantly higher adiponectin levels in T1D patients (13.5 ± 1.0 μg/mL) compared to controls (8.8 ± 1.0 μg/mL, *p* < 0.0001) [25]. These elevated levels positively correlated with HDL cholesterol and inversely with body mass index (BMI) and markers of central adiposity [25]. The study further showed that adiponectin levels in T1D were independently associated with hemoglobin A1c (HbA1c) and albumin excretion rate [25]. Similarly, Frystyk et al. reported serum adiponectin levels of 23.7 ± 14.3 mg/L in T1D patients, compared to 13.5 ± 4.7 mg/L in controls (*p* < 0.0001), and these elevated levels may reflect a response to microvascular complications [26]. The study found higher adiponectin levels in T1D with proliferative retinopathy compared to those with no or non-proliferative retinopathy, and in patients with nephropathy compared to normoalbuminuric individuals [26]. These findings suggest that elevated adiponectin in T1D may serve as a counter-regulatory response to chronic inflammation and vascular stress in microvascular complications [26].

Further research has confirmed that adiponectin levels are elevated in individuals with T1D, with increases ranging from 28% to 98% compared to healthy controls [5, 25, 26]. This elevation is thought to result from insulin deficiency, loss of pancreatic beta-cell function, and possible tissue resistance to adiponectin [1, 5, 25, 26]. Normally, insulin suppresses adiponectin secretion in a dose- and time-dependent manner, so low insulin levels in T1D could drive increased adiponectin secretion. Our recent study supports this idea, showing that, even after adjusting for diabetes-related variables (e.g., HbA1c, C-peptide, insulin resistance), the total daily insulin dose was inversely correlated with serum adiponectin levels [2]. This suggests that low insulin levels in T1D contribute to elevated adiponectin concentrations. Additionally, chronic hyperglycemia, oxidative stress, and low-grade inflammation in T1D may alter adiponectin’s three-dimensional structure, potentially reducing receptor affinity and causing resistance to its effects [3, 27, 28]. Combs et al. explored this phenomenon in a comparative study involving non-obese diabetic (NOD) mice and human participants with T1D [29]. The study focused on the relationship between adiponectin and SOGA (Suppressor of Glucose from Autophagy), a liver-derived protein critical for glucose metabolism [29]. Adiponectin stimulates SOGA production in hepatocytes, and SOGA fragments are used as markers of adiponectin signaling activity in the liver [29]. Interestingly, while serum adiponectin levels in T1D patients were 50%–60% higher than in controls, circulating SOGA levels were 30%–50% lower [29]. Additionally, glucose appearance under low insulin infusion negatively correlated with both adiponectin and SOGA [29]. These findings suggest a dissociation in adiponectin signaling, supporting the hypothesis of tissue resistance to adiponectin in T1D.

Overall, elevated adiponectin levels in T1D likely represent a complex interplay of insulin deficiency, tissue resistance, and compensatory metabolic adaptation. While these levels may initially reflect a protective response, the presence of signaling defects and receptor resistance diminishes their beneficial effects, particularly in managing glucose metabolism and preventing complications (Fig. 1).

### Adiponectin in Type 2 Diabetes

In contrast to T1D, adiponectin levels are typically reduced in T2D due to several interconnected mechanisms. T2D patients retain some beta-cell function, resulting in C-peptide levels comparable to those of non-diabetic individuals [1, 14, 16, 30]. This residual beta-cell activity, combined with insulin resistance, suppresses adiponectin secretion. Intensive insulin therapy in T2D patients has been shown to increase adiponectin levels by up to 15%, accompanied by a significant reduction in C-reactive protein (CRP), though the effect was not consistently linked to treatment duration [30].

Adiponectin levels in T2D patients are typically 30–50% lower than in healthy individuals, with this reduction also correlating with higher visceral fat mass (*r* = −0.57, *P* < 0.001) [1]. Insulin resistance, a hallmark of T2D, impairs adipose tissue function, the primary source of adiponectin production. Dysfunctional adipose tissue reduces adiponectin secretion, while inflammation and ectopic fat accumulation further suppresses its levels [1]. Chronic hyperglycemia and oxidative stress, both characteristics of T2D, impair adipocyte function. In fact, oxidative stress markers inversely correlate with adiponectin levels (*r* = −0.45, *P* < 0.01) and increase the risk of insulin resistance (*OR* = 4.1, *P* < 0.001) [31]. Pro-inflammatory cytokines, such as TNF-α and IL-6, contribute to this reduced adiponectin secretion. Elevated TNF-α, for instance, reduces adiponectin expression in adipose tissue by 20–30%, with a significant association between inflammation and decreased insulin sensitivity (*OR* = 3.5, *P* = 0.002) [15]. Additionally, elevated free fatty acids (FFAs), common in insulin-resistant states, inhibit adiponectin gene expression and secretion. Higher FFAs have been associated with a 25% reduction in adiponectin secretion and an increased odds ratio (*OR* = 2.8, *P* < 0.01) for developing T2D [32]. Visceral adipose tissue in insulin-resistant individuals shows approximately 35–40% lower adiponectin production compared to subcutaneous fat, further contributing to metabolic dysfunction (*OR* = 3.2, *P* < 0.01) [10].

Receptor dysfunction also plays a critical role in adiponectin resistance. A study reported a 30% reduction in AdipoR1 signaling in diabetic patients, mainly due to phosphorylation-induced receptor impairment, along with a 50% reduction in downstream anti-inflammatory signaling pathways [3]. Levels of HMW adiponectin, the most biologically active isoform, were significantly lower in diabetic individuals compared to healthy controls (10 μg/mL vs. 20 μg/mL) [3]. Similarly, AdipoR2 expression in the liver is markedly lower in insulin-resistant individuals, contributing to increased triglyceride accumulation and worsening lipid dysregulation [3]. Visceral adiposity, a major driver of adipokine imbalance, is strongly associated with decreased adiponectin levels (*r* = −0.57, *P* < 0.001), which increases the likelihood of metabolic dysfunction [6, 14]. As weight gain progresses, adipose tissue homeostasis is disrupted by hypoxia, oxidative stress, inflammation, and fibrosis, which lead to imbalances in adipokine secretion and a further decline in adiponectin expression [6].

In summary, reduced adiponectin levels in T2D are largely driven by insulin resistance, dysfunctional adipose tissue, inflammation, and receptor dysfunction. These factors contribute to impaired metabolic control, including the worsening of insulin resistance and cardiovascular complications (Fig. 1).

## Cardiovascular Effects of Adiponectin

Adiponectin exerts significant cardioprotective effects through its anti-inflammatory, anti-apoptotic, and lipid-modulating properties. By activating the AMPK and PPARα signaling pathways, adiponectin promotes fatty acid oxidation, reduces oxidative stress, and enhances endothelial function [33]. These mechanisms collectively help reduce the risk of atherosclerosis, prevent myocardial remodeling, and mitigate cardiovascular complications associated with diabetes and other metabolic disorders. Through its protective actions, adiponectin plays a crucial role in maintaining cardiovascular health, particularly in individuals with metabolic diseases like diabetes.

### Protective Roles

#### Endothelial Function and Vascular Health

Endothelial dysfunction is a key feature of cardiovascular disease, often characterized by reduced nitric oxide (NO) bioavailability and increased oxidative stress [34]. Adiponectin plays a vital role in improving endothelial function by enhancing endothelial nitric oxide synthase (eNOS) activity, which boosts NO production, reduces vascular stiffness, and promotes vasodilation [34]. A clinical study demonstrated that adiponectin increased NO bioavailability by 30% (*P* < 0.01), highlighting its protective role in maintaining endothelial function. Additionally, adiponectin inhibits vascular smooth muscle cell (VSMC) proliferation and migration, both of which are crucial in the development of neointimal hyperplasia and restenosis, contributing to vascular remodeling and atherosclerosis [35].

#### Anti-Atherogenic Properties

Adiponectin is also crucial in preventing atherosclerosis by reducing the formation of foam cells, a key step in early atherogenesis. It suppresses the uptake of oxidized low-density lipoprotein (oxLDL) by macrophages, thereby limiting lipid deposition in arterial walls [36]. In a study with atherosclerosis-prone mice, adiponectin therapy reduced total plaque size by 40% (*P* < 0.001) and decreased macrophage infiltration by 35% (*P* < 0.01), underscoring its role in reducing vascular inflammation. Furthermore, adiponectin promotes reverse cholesterol transport by upregulating the ATP-binding cassette transporter A1 (ABCA1), facilitating cholesterol efflux from macrophages and helping clear cholesterol from plaques [37]. Clinical data have also shown that a 10 μg/mL increase in adiponectin was associated with a 0.13 mmol/L increase in HDL cholesterol (*P* < 0.001), linking adiponectin to improved cholesterol metabolism and reduced cardiovascular risk [38].

#### Prevention of Myocardial Remodeling

Adiponectin plays a protective role in the myocardium by reducing inflammation, oxidative stress, and lipid accumulation, all of which are critical contributors to myocardial remodeling after conditions like myocardial infarction (MI) and diabetic cardiomyopathy. Through AMPK activation, adiponectin enhances mitochondrial function, promotes fatty acid oxidation, and prevents lipotoxicity in cardiomyocytes [1, 6, 34]. It also inhibits TNF-α and NF-κB activation, which are key mediators of inflammation-induced myocardial damage [39, 40]. In a post-MI study, higher adiponectin levels were associated with a 23% lower risk of left ventricular dysfunction (*HR* = 0.77; 95% CI: 0.62–0.95), indicating its role in preventing adverse cardiac remodeling. Experimental MI models have shown that adiponectin therapy improves left ventricular ejection fraction (LVEF) by 15% (*P* < 0.05) and reduces myocardial fibrosis by 25% [40]. Additionally, adiponectin reduces cardiomyocyte apoptosis by modulating pathways like sphingosine kinase-1/COX-2 signaling, protecting against cell death during ischemia-reperfusion injury [40]. Patients with high adiponectin levels (>12 μg/mL) were found to have a 32% lower risk of ischemic cardiomyopathy progression (*OR* = 0.68; 95% CI: 0.51–0.89), reinforcing its protective effects on the heart [41].

#### Systemic Effects on Cardiovascular Risk

Adiponectin’s systemic effects extend beyond the heart, reducing cardiovascular risk through its influence on lipid metabolism and inflammation. In human aortic endothelial cells (HAECs), adiponectin activates AMPK, which suppresses NF-κB signaling, ultimately decreasing CRP synthesis and secretion under hyperglycemic conditions. CRP is a key biomarker of vascular inflammation and plays a significant role in atherogenesis, linking adiponectin to reduced cardiovascular risk [42]. Further, a study by Kazumi et al. found that higher adiponectin levels were significantly correlated with increased HDL cholesterol (*r* = 0.37, *P* < 0.01) and decreased triglyceride levels (*r* = −0.25, *P* < 0.05) [43]. A prospective cohort study by Lindberg et al. followed 5349 participants over 8.5 years and found that higher adiponectin levels were associated with a significantly lower risk of cardiovascular events. Specifically, each doubling of plasma adiponectin resulted in a hazard ratio (HR) of 0.34 (95% CI: 0.16–0.72, *P* = 0.005), highlighting its potential protective role in cardiovascular health [44].

### Adiponectin Paradox

Adiponectin is generally recognized for its cardioprotective effects, but its role in metabolic and cardiovascular health becomes more complex in the context of **T1D** and **T2D.** The **adiponectin paradox** refers to the puzzling observation that, while elevated adiponectin levels are typically associated with protection against metabolic and cardiovascular diseases, they paradoxically correlate with increased cardiovascular and all-cause mortality in certain populations, particularly those with diabetes. Though low adiponectin levels are linked to insulin resistance, obesity, and atherosclerosis, high adiponectin levels are often found in individuals with advanced cardiovascular conditions, such as heart failure (HF), where they fail to exert protective effects and may even signal worse outcomes.

#### Adiponectin Paradox in Type 1 Diabetes

In T1D, elevated adiponectin levels have been paradoxically linked to both protective and harmful cardiovascular outcomes. While adiponectin’s anti-inflammatory effects provide protection in the early stages of the disease, studies suggest that higher levels (greater than 15 μg/mL) are associated with an increased risk of cardiovascular complications and all-cause mortality [2, 5]. For example, our study found that T1D patients with elevated adiponectin levels were 40% more likely to experience cardiovascular events compared to those with moderate levels [2]. While most research associates higher adiponectin levels with negative outcomes in T1D, some studies show exceptions. Costacou et al. reported that higher adiponectin levels were linked to a 63% reduction in coronary artery disease (CAD) risk (HR: 0.37, 95% CI: 0.19–0.73, *p* = 0.004) in patients with macroalbuminuria [45]. This subgroup had significantly higher adiponectin levels (21.3 μg/mL vs. 17.4 μg/mL; *p* = 0.04) than those without macroalbuminuria, indicating that elevated adiponectin may have a context-specific protective role in T1D [45]. However, this protective effect may be limited to certain subgroups and is likely outweighed by adiponectin resistance in advanced cardiovascular and inflammatory conditions [45].

#### Adiponectin Paradox in Type 2 Diabetes

In T2D, the “adiponectin paradox” underscores the surprising link between elevated adiponectin levels and increased cardiovascular and all-cause mortality. Tu et al. explored adiponectin levels in 4274 patients with acute ischemic stroke over a three-year period and found significant associations between higher adiponectin levels and an increased risk of major adverse cardiovascular and cerebrovascular events (MACE) and mortality [46]. Patients in the highest adiponectin quartile (Q4, >9.8 μg/mL) had a 4.95-fold higher risk of MACE (HR: 4.95, 95% CI: 3.03–7.06) and a 5.63-fold higher risk of all-cause mortality (HR: 5.63, 95% CI: 3.15–7.99) compared to those in the lowest quartile (Q1, <4.8 μg/mL) [46]. These risks remained significant even after adjusting for traditional confounders like age, BMI, and CRP levels [46].

Francischetti et al. further emphasized that circulating adiponectin levels above 20 μg/mL were associated with a 30% increase in all-cause mortality among individuals with T2D [47]. In contrast, individuals with adiponectin levels in the normal range (3–10 μg/mL) had significantly lower mortality risks. The study also highlighted adiponectin resistance, noting a 50% reduction in AdipoR1 receptor activity in patients with metabolic syndrome [47]. Singer et al. also confirmed that T2D patients in the highest adiponectin quartile (>20 μg/mL) had a hazard ratio (HR) of 4.0 (95% CI: 1.7–9.2) for all-cause mortality compared to those in the lowest quartile, even after adjusting for confounders like medications and cardiovascular risk factors [48]. Similarly, Menzaghi et al. found that excessively high adiponectin levels (>15 μg/mL) were linked to worse outcomes, including a HR of 1.32 for cardiovascular mortality when levels exceeded 20 μg/mL [20]. These findings suggest that beyond a certain threshold, adiponectin’s effects shift from protective to potentially harmful [20].

## Adiponectin in Heart Failure

In heart failure (HF), adiponectin levels are often significantly elevated and correlate with the severity of the condition. These increased levels are paradoxically associated with heightened mortality [20]. The Heart and Soul Study found that patients with ischemic HF in the highest adiponectin quartile had a 67% higher risk of mortality (HR: 1.67, 95% CI: 1.24–2.26) and a 63% higher risk of HF hospitalization (HR: 1.63, 95% CI: 1.04–2.56) [49]. Jang et al. further explored the interaction between adiponectin and different heart failure phenotypes [50]. In heart failure with reduced ejection fraction (HFrEF), elevated adiponectin levels were linked to higher natriuretic peptide (NP) secretion, contributing to metabolic adaptations that could not compensate for systemic resistance [50]. In contrast, heart failure with preserved ejection fraction (HFpEF) displayed lower NP levels, which were associated with greater myocardial stiffness and inflammation [50]. This pattern, observed in both ischemic and non-ischemic HF, reflects a compensatory response to metabolic and cardiovascular stress but also serves as a marker of poor prognosis [2, 50–52]. The Physicians’ Health Study also revealed a U-shaped association between total adiponectin levels and HF incidence among U.S. male physicians [53]. In this prospective nested case-control study, the risk of HF varied non-linearly across quintiles of adiponectin concentrations. The adjusted relative risks across the quintiles (from lowest to highest) were 1.00 (ref), 0.74 (95% CI: 0.53–1.04), 0.67 (95% CI: 0.48–0.94), 0.70 (95% CI: 0.50–0.99), and 0.92 (95% CI: 0.65–1.30), with all higher quintiles suggesting a protective association and the third quintile showing the lowest risk of HF [53]. This complex relationship suggests that moderate levels of adiponectin may provide cardioprotective effects, while both very high and very low levels are linked to increased HF risk (Fig. 1).

### Adiponectin Resistance in Heart Failure

Adiponectin resistance plays a crucial role in HF, where elevated circulating adiponectin levels fail to confer protective effects and instead correlate with worse cardiovascular outcomes. This resistance results from several factors, including receptor desensitization, systemic inflammation, ventricular stress, and oxidative damage. These conditions impair adiponectin’s ability to regulate glucose metabolism, reduce oxidative stress, and maintain lipid homeostasis. Additionally, structural remodeling of adiponectin receptors, such as glycosylation and hydroxylation under hyperglycemic conditions, reduces receptor affinity and disrupts downstream signaling, thereby exacerbating HF progression [2].

Khan et al. demonstrated that patients with advanced HF exhibited increased adipose tissue inflammation, marked by higher macrophage infiltration and reduced adiponectin receptor signaling [54]. This systemic inflammation contributed to adiponectin resistance, which partially normalized after ventricular assist device (VAD) implantation. Specifically, adiponectin levels decreased significantly (from 13.3 ± 4.9 μg/mL pre-VAD to 7.4 ± 3.4 μg/mL post-VAD; *p* < 0.05), which was associated with improvements in insulin resistance and cardiac function [54]. Sharma et al. reported a 60% reduction in AdipoR1 and AdipoR2 expression in cardiac tissues from HF patients with T2D [9]. This downregulation of receptors impaired AMP-activated protein kinase (AMPK) activation, leading to a 40% reduction in mitochondrial efficiency, glucose uptake, and oxidative stress defenses [9]. As a result, adiponectin’s anti-inflammatory effects were diminished, contributing to further deterioration in HF [9]. Similarly, Matsuda et al. highlighted that receptor dysfunction limits fatty acid oxidation and lipid clearance, further worsening metabolic dysregulation in HF [11]. Jang et al. emphasized that adiponectin resistance goes beyond systemic effects, extending to maladaptive cardiac remodeling [50]. Their findings showed that receptor-level impairments in HF patients reduced AMPK activation, which further diminished adiponectin’s potential to protect the heart [50].

### Structural and Functional Changes in Heart Failure

Adiponectin resistance in HF is closely associated with structural and functional cardiac remodeling, where elevated adiponectin levels often reflect maladaptive responses to chronic stress. These structural changes include left ventricular (LV) hypertrophy, increased left ventricular end-diastolic volume (LVEDV), and left atrial (LA) remodeling, all of which contribute to worsening cardiac function [2, 55, 56]. Our previous study demonstrated significant correlations between elevated adiponectin levels and increased LVEDV, a key marker of adverse cardiac remodeling [2]. Patients in the highest adiponectin quartile had significantly higher LVEDV indices, indicating increased wall stress and reduced cardiac efficiency. Additionally, LV mass-to-volume ratios were lower in patients with higher adiponectin levels (0.98 ± 0.14 vs. 1.07 ± 0.17 in the lowest quartile), signaling myocardial remodeling associated with impaired cardiac performance [2]. Won et al. provided further evidence of the connection between adiponectin levels and cardiac dysfunction, showing that adiponectin levels in HF patients were correlated with markers of diastolic dysfunction and structural changes [55]. Plasma adiponectin levels were significantly lower in patients with metabolic syndrome (9.7 ± 7.0 μg/mL) compared to those without metabolic syndrome (15.8 ± 10.9 μg/mL, *p* = 0.001) [55]. Despite these lower levels, higher adiponectin concentrations were associated with worse cardiac function, including elevated left atrial volume index (LAVI, *r* = 0.197, *p* = 0.026) and increased E/E’ ratios (*r* = 0.217, *p* = 0.021), both reflecting impaired diastolic performance [55].

### BNP-Adiponectin Interplay

Natriuretic peptides (NPs), particularly brain natriuretic peptides (BNP), play a pivotal role in the resistance to adiponectin and are central to understanding its paradoxical effects in HF. BNP levels rise in response to increased ventricular stress in HF, promoting natriuresis and vasodilation. However, BNP also stimulates adiponectin production, which paradoxically exacerbates adiponectin resistance in advanced HF. Tamura et al. demonstrated that chronic HF patients had significantly higher serum adiponectin levels (14.6 μg/mL) compared to controls (6.7 μg/mL, *p* < 0.0001), with a positive correlation between BNP and adiponectin levels (*r* = 0.48, *p* = 0.005) [57]. Takano et al. explored the role of adiponectin in HF progression, showing that adiponectin secretion from the myocardium contributes to its elevated circulating levels during disease progression [56]. Tsukamoto et al. further emphasized this interaction, showing that chronic HF patients receiving atrial natriuretic peptide (ANP) experienced a significant increase in adiponectin levels (from 5.66 ± 0.40 μg/mL to 7.34 ± 0.47 μg/mL; *p* = 0.05), highlighting the role of NPs in regulating adiponectin production through the cGMP-PKG signaling pathway [58]. This interplay suggests that while NPs aim to improve mitochondrial function and mitigate cardiac stress, they also contribute to adiponectin resistance, compounding the challenges in managing HF.

Baldasseroni et al. examined the complex relationship between BNP and adiponectin in chronic HF, showing that although BNP elevated adiponectin levels, this did not translate into improved metabolic or inflammatory outcomes [59]. Their study highlighted that non-diabetic HF patients exhibited a steady increase in adiponectin levels as HF severity progressed, from 10.7 ± 9.3 ng/mL in early HF to 15.3 ± 8.8 ng/mL in advanced HF [59]. In contrast, diabetic HF patients had significantly lower adiponectin levels (7.9 ± 5.0 ng/mL), with increases only observed in later stages of the disease. Despite similar BNP levels between diabetic and non-diabetic patients, these findings suggest that diabetes may impair the compensatory adiponectin response, potentially through mechanisms like “BNP resistance” [59]. This points to the intricate relationship between BNP, adiponectin, and metabolic dysfunction in HF, underscoring the role of the adiponectin paradox.

## Clinical Implications and Future Directions

### Adiponectin and BNPs in Risk Stratification

Elevated BNP levels, commonly used as markers of HF severity, are closely linked with adiponectin levels, particularly in individuals with T1D and T2D. This correlation has important implications for predicting HF outcomes. Karas et al. identified a threshold effect in older HF patients, where total adiponectin levels >12.4 μg/mL significantly increased the risk of incident HF (HR: 1.25, 95% CI: 1.14–1.38) [18]. Kistorp et al. also linked high adiponectin levels to worse outcomes in HF patients, particularly among those with T2D. Their study found that patients with adiponectin levels >20 μg/mL faced a higher risk of mortality (HR: 1.30, 95% CI: 1.14–1.48) [60].

Additionally, Allison et al. demonstrated that a 1-SD increase in adiponectin was associated with a 25 pg/mL rise in BNP [61]. Participants in the highest adiponectin quartile had a 67% increase in BNP levels [61]. However, the interplay between adiponectin and BNP complicates their interpretation as isolated biomarkers, as their combined effects may provide more valuable insights into disease status. Lindberg et al. conducted a prospective study of 5574 individuals without ischemic heart disease or HF, finding that higher plasma adiponectin levels were associated with an increased risk of HF (HR 1.20 per 1 SD increase, 95% CI 1.06–1.30; *P* = 0.003). However, this association lost statistical significance (HR 1.08, 95% CI 0.95–1.23; *P* = 0.26) after adjusting for BNP, suggesting that BNP may act as a strong confounder in the relationship between adiponectin and HF risk [51].

Zhao et al. explored the adiponectin-to-leptin ratio as an additional predictive factor for cardiovascular events in patients with obesity-related disorders. They reported that a ratio below 1 was associated with a 25% higher risk of cardiovascular events, while increasing the ratio above 1.5 led to a significant reduction in inflammatory markers (approximately 30%) and improvements in endothelial function [46]. These findings suggest that the adiponectin-to-leptin ratio could be an important therapeutic target, helping to mitigate systemic inflammation and improve cardiovascular health by improving endothelial function.

### AdipoR1 and AdipoR2: Promising Therapeutic Targets

The role of AdipoR1 and AdipoR2 in mediating adiponectin’s effects makes them highly attractive targets for therapeutic interventions. Enhancing receptor sensitivity or expression could potentially restore adiponectin signaling and ameliorate the metabolic and cardiovascular dysfunction observed in conditions like HF and diabetes. Experimental therapies, such as small molecules like AdipoRon, a synthetic agonist of adiponectin receptors, have shown promising results in preclinical models. AdipoRon activates both AdipoR1 and AdipoR2 pathways, and studies have demonstrated its ability to improve glucose tolerance, reduce liver triglycerides, and enhance AMPK activation in models of T2D [3, 8, 62]. These findings suggest that AdipoRon could play a crucial role in the treatment of HF, particularly in patients with metabolic comorbidities such as diabetes, by restoring adiponectin’s beneficial effects on metabolism and heart function.

In addition to receptor agonists like AdipoRon, other therapeutic strategies focus on addressing the underlying mechanisms of adiponectin resistance, such as inflammation and oxidative stress. These approaches aim to preserve receptor function by counteracting the negative effects of systemic inflammation. For example, antioxidant therapies that reduce NF-κB activation could help maintain receptor expression and improve adiponectin’s ability to regulate metabolism and cardiovascular health [47]. By targeting these mechanisms, it may be possible to reduce adiponectin resistance in HF and diabetes, thus preserving the protective effects of adiponectin.

### Limitations and Future indications

Despite significant insights into the role of adiponectin in diabetes and heart failure, several limitations still impede a comprehensive understanding. A key challenge lies in the variability among study populations, including differences in age, sex, and diabetes duration, which complicates the generalizability of findings. Additionally, further research is needed to explore sex-specific differences in adiponectin biology. Women generally exhibit higher levels of HMW adiponectin, which is linked to better metabolic outcomes. Examining the influence of hormonal factors, such as estrogen, on adiponectin receptor sensitivity could offer valuable insights into its role in cardiovascular and metabolic diseases, potentially leading to targeted therapies that harness hormonal modulation to enhance adiponectin’s protective effects.

The intricate interplay between adiponectin and BNPs presents additional challenges, limiting their reliability as biomarkers. A promising direction for future clinical application is the integration of these biomarkers into risk stratification tools. Research should aim to establish specific adiponectin thresholds that, when combined with BNP measurements, can differentiate between HF phenotypes such as HFpEF and HFrEF. This approach could improve the predictive accuracy of these biomarkers and support more personalized patient care. Expanding on these findings, the inclusion of multi-marker panels incorporating adiponectin and BNPs could further optimize HF management strategies. Longitudinal studies tracking adiponectin levels over time, in relation to disease progression and therapeutic interventions, are essential to clarify whether adiponectin’s role is compensatory or pathological.

Therapies like AdipoRon, which activate adiponectin receptors, present a promising approach for managing HF, particularly in patients with metabolic comorbidities such as diabetes and obesity. Future research should explore the combined use of adiponectin-modulating therapies with anti-inflammatory treatments to address both the metabolic and cardiovascular components of HF. A multi-targeted strategy that integrates BNP-based interventions alongside adiponectin modulation could lead to more effective management, especially for patients with diabetes-related cardiomyopathy. To further advance the field, it is essential to address mechanistic gaps, including the underlying triggers of hyperadiponectinemia and adiponectin resistance. Additionally, investigating structural modifications of adiponectin, such as glycosylation and hydroxylation, and their impact on receptor binding and downstream signaling will be crucial. These insights could reveal novel therapeutic targets aimed at restoring adiponectin’s cardioprotective effects.

In addition, the relationship between adiponectin and HF in T1D remains underexplored. While some studies have linked elevated adiponectin with left ventricular remodeling [2, 63], the precise mechanisms through which insulin deficiency and chronic hyperglycemia influence HF progression in T1D need further investigation. Understanding whether adiponectin elevation in T1D is compensatory or pathological could lead to targeted therapeutic strategies.

Ultimately, a deeper understanding of adiponectin’s complex role in HF and diabetes will require a multidisciplinary approach. This entails improving the generalizability of findings, refining adiponectin’s potential as both a biomarker and a therapeutic target, addressing mechanistic gaps, and developing novel therapies that modulate adiponectin signaling. Integrating adiponectin and BNPs into risk stratification tools, alongside a more comprehensive exploration of adiponectin resistance and its underlying mechanisms, could pave the way for more effective and personalized management strategies, particularly for patients with HF and diabetes.

## Conclusion

Adiponectin plays a vital role in diabetes, influencing key metabolic processes such as insulin sensitivity, lipid metabolism, inflammation, and oxidative stress. In T1D, elevated adiponectin levels often serve as a compensatory mechanism to address beta-cell dysfunction and maintain metabolic balance. In contrast, T2D is characterized by reduced adiponectin levels, which are closely linked to insulin resistance, visceral adiposity, and chronic inflammation. Through its receptors, AdipoR1 and AdipoR2, adiponectin not only tackles these metabolic challenges but also extends its protective effects to the cardiovascular system, making it a crucial factor in both metabolic and heart health.

Beyond diabetes, adiponectin also plays a significant, multifaceted role in HF. It helps protect against HF progression by suppressing inflammation, promoting fatty acid oxidation, and enhancing endothelial function. However, in advanced HF, high adiponectin levels, known as hyperadiponectinemia, often emerge as a compensatory response to systemic stress, reflecting the body’s efforts to counterbalance the failing heart’s demands. Additionally, adiponectin’s interactions with other biomarkers, such as BNPs, highlight the importance of developing refined risk stratification tools to personalize patient care and improve outcomes in HF management.

The growing understanding of adiponectin’s dual role in metabolic and cardiovascular health opens exciting therapeutic possibilities. By modulating insulin sensitivity and reducing inflammation, adiponectin offers a promising target for addressing diabetes-related cardiovascular risks. Future treatments could focus on balancing its protective and maladaptive effects, especially when combined with biomarkers like BNPs for more effective care. This approach has the potential to transform the management of diabetes-related HF, paving the way for personalized and comprehensive treatment strategies for diverse patient populations.

## Key References

- Mao, Y., & Zhong, W. (2023). Serum adiponectin concentrations as a risk factor for cardiovascular complications in type 1 diabetes. *Diabetes Research and Clinical Practice*, *200*. 10.1016/j.diabres.2023.110700
  - Explores the protective role of adiponectin in carotid atherosclerosis and peripheral artery disease in type 1 diabetes, while also examining its potential association with increased cardiovascular events depending on cardiac structural changes.
- Sharma, A., Mah, M., Ritchie, R. H., & de Blasio, M. J. (2022). The adiponectin signaling pathway - A therapeutic target for the cardiac complications of type 2 diabetes? In *Pharmacology and Therapeutics* (Vol. 232). Elsevier Inc. 10.1016/j.pharmthera.2021.108008
  - Investigate the adiponectin signaling pathway in diabetic cardiomyopathy, highlighting its role in regulating myocardial metabolism, inflammation, and oxidative stress, with implications for targeted therapeutic interventions in type 2 diabetes.
- Han, Y., Sun, Q., Chen, W., Gao, Y., Ye, J., Chen, Y., Wang, T., Gao, L., Liu, Y., & Yang, Y. (2024). New advances of adiponectin in regulating obesity and related metabolic syndromes. In *Journal of Pharmaceutical Analysis* (Vol. 14, Issue 5). 10.1016/j.jpha.2023.12.003
  - Provides an updated analysis of the role of adiponectin in obesity and metabolic syndromes, discussing its regulatory mechanisms and potential therapeutic applications in metabolic disorders.
- Jang, A. Y., Scherer, P. E., Kim, J. Y., Lim, S., & Koh, K. K. (2022). Adiponectin and cardiometabolic trait and mortality: where do we go? In *Cardiovascular Research* (Vol. 118, Issue 9, pp. 2074–2084). Oxford University Press. 10.1093/cvr/cvab199
  - Examines the complex interplay between adiponectin, cardiometabolic traits, and mortality, synthesizing current evidence to evaluate its potential as a prognostic biomarker. It highlights key challenges and future directions in understanding how adiponectin may influence cardiovascular risk and overall mortality, offering a roadmap for future research in the field.
- Aljafary, M. A., & Al-Suhaimi, E. A. (2022). Adiponectin System (Rescue Hormone): The Missing Link between Metabolic and Cardiovascular Diseases. In *Pharmaceutics* (Vol. 14, Issue 7). MDPI. 10.3390/pharmaceutics14071430
  - Provides a comprehensive yet succinct overview of the adiponectin system as a key regulatory axis linking metabolic dysfunction and cardiovascular disease. It highlights adiponectin’s role in lipid metabolism, insulin sensitivity, and vascular homeostasis, while discussing its therapeutic potential in mitigating metabolic and cardiovascular complications.
- Zhou, Z., Liu, C., Xu, S. et al. Metabolism regulator adiponectin prevents cardiac remodeling and ventricular arrhythmias via sympathetic modulation in a myocardial infarction model. *Basic Res Cardiol* **117**, 34 (2022). 10.1007/s00395-022-00939-2
  - Investigates adiponectin’s role in preventing adverse cardiac remodeling and ventricular arrhythmias following myocardial infarction. It highlights adiponectin’s impact on sympathetic regulation as a potential mechanism for cardioprotection.

## Data Availability

No datasets were generated or analyzed during the current study.
